# Intensification of Polyphenols Extraction from *Eryngium creticum* Leaves Using *Ired-Irrad*^®^ and Evaluation of Antibiofilm and Antibacterial Activities

**DOI:** 10.3390/plants11192458

**Published:** 2022-09-21

**Authors:** Mariam Hammoud, Ali Chokr, Hiba N. Rajha, Carl Safi, Martijn van Walsem, Lambertus A. M. van den Broek, Espérance Debs, Richard G. Maroun, Nicolas Louka, Hassan Rammal

**Affiliations:** 1 Research Laboratory of Microbiology (RLM), Department of Life and Earth Sciences, Faculty of Sciences I, Lebanese University, Hadat Campus, P.O. Box 5, Beirut 1683, Lebanon; 2 Centre d’Analyses et de Recherche, Unité de Recherche Technologies et Valorisation Agro-Alimentaire, Faculté des Sciences, Université Saint-Joseph de Beyrouth, Riad El Solh, P.O. Box 17-5208, Beirut 1104 2020, Lebanon; 3 Platform of Research and Analysis in Environmental Sciences (PRASE), Doctoral School of Sciences and Technology (DSST), Lebanese University, Hadat Campus, P. O. Box 6573/14, Beirut 1683, Lebanon; 4 Ecole Supérieure d’Ingénieurs de Beyrouth (ESIB), Université Saint-Joseph de Beyrouth, CST Mkalles Mar Roukos, Riad El Solh, P.O. Box 11-514, Beirut 1107 2050, Lebanon; 5 Wageningen Food & Biobased Research, P.O. Box 17, 6700 AA Wageningen, The Netherlands; 6 Department of Biology, Faculty of Arts and Sciences, University of Balamand, P.O. Box 100, Tripoli 1300, Lebanon; 7 Faculty of Agronomy, Lebanese University, Dekweneh, Beirut 1683, Lebanon

**Keywords:** antibacterial activity, antibiofilm activity, *Eryngium creticum*, *Ired-Irrad^®^*, polyphenols

## Abstract

(1) Background: *Eryngium creticum* is a plant medicinally valued, and used in pharmacopeia to treat various diseases. No previous studies have been reported on *E. creticum* leaf extracts using an IR-assisted technique; thus, this study aimed to intensify polyphenol extraction using *Ired-Irrad^®^*, comparing it to the conventional water bath (WB) method. (2) Methods: Optimization of polyphenol extraction from *E. creticum* leaves was conducted using Response Surface Methodology. *Ired-Irrad^®^* was used and compared to the WB method. The biological activities (antiradical, antioxidant, antibacterial, and antibiofilm) of both extracts were assessed. UHPLC analysis was performed to analyze the phytochemical profile of both extracts. (3) Results: Under optimal conditions, IR improved the polyphenol extraction yield by 1.7 times, while lowering ethanol consumption by 1.5 times. Regarding the antibacterial activity, both WB and IR *E. creticum* leaf extracts exhibited the highest antibacterial activity against *Staphylococcus epidermidis*. The maximum biofilm prevention capacity was also noticed against *S. epidermidis*. UHPLC-MS analysis quantified two major phenolic compounds in both extracts: rutin and sinapic acid. (4) Conclusions: *Ired-Irrad^®^* technology proved to be an effective technique in intensifying polyphenol recovery, while preserving their quantity and quality.

## 1. Introduction

*Eryngium creticum*, a perennial plant belonging to the Apiaceae family, is found in East Mediterranean countries such as Lebanon, Palestine, Jordan, and Syria where it is consumed in salads. This plant, known as Kors-Anneh, is valued medicinally and used in pharmacopeia for the treatment of many diseases such as liver diseases, anemia, and infertility [1]. The infusion of roots and seeds of this plant is also recommended to treat kidney stones, skin infections, and tumors. *E. creticum* is considered an alternative to an antidote since it may be beneficial in the treatment of snakebites and scorpion stings [2]. Previous studies carried out on biological activities of *E. creticum* have confirmed its antioxidant, anti-inflammatory, and antimicrobial properties. Other studies reported the presence of terpenoids, saponins, flavonoids, coumarin, polyacetylenes, steroids, and essential oils in the *Eryngium* genus [3,4,5]. Nevertheless, most species of *Eryngium* have not yet been explored for their chemical composition.

Phenolic compounds, the largest group of plant secondary metabolites, are abundant in fruits, vegetables, and herbal teas, and have been exhaustively investigated due to their beneficial effects on human health and their use as food additives [6]. Therefore, different techniques were developed aiming to extract polyphenols from plant materials. Although the conventional water bath method (WB) presents many disadvantages such as the high consumption of energy and of organic solvents, it is still widely used due to its simplicity [7]. Ultrasound-assisted extraction (UAE), microwave-assisted extraction (MAE), pressurized liquid extraction (PAE), supercritical fluid extraction (SFE), pulsed-electric field extraction (PEF), high-voltage electrical discharges (HVED), and other extraction methods are known as being non-conventional. These novel techniques offer several advantages such as shorter extraction times, reduced organic solvent consumption, and higher extraction yields [8,9].

*Ired-Irrad^®^* (IR) is a patented extraction technique [10] that uses the infrared energy released from a ceramic emitter to heat the solvent–matrix mixture [11]. This treatment has been proven to improve the extraction yield of phenolic compounds [12] from different plant materials such as pomegranate peels [13], orange peels [14], and olive leaves [11] compared to conventional extraction methods. Regarding the quality of these IR extracts, they exhibited a higher antiradical activity, which could be related to the reduced extraction time applied on one hand, and to the mode of action (heat transfer) of infrared irradiations on the other hand.

To our knowledge, no previous studies have been reported on polyphenol extraction from *E. creticum* leaves using an IR-assisted technique. Hence, the aim of this study was to enhance polyphenol extraction from *E. creticum* leaves using *Ired-Irrad^®^*, to identify and quantify the phenolic compounds, and to evaluate the antioxidant, antibacterial, and antibiofilm capacities of the leaf extracts.

## 2. Results

### 2.1. Selection of Particle Size and Solid to Liquid Ratio

The determination of particle size was based on preliminary experiments. The reduction in particle size from a whole leaf to 2–4.75 mm increased significantly (*p* < 0.05) the polyphenols content of the extract from 15 to 25 mg Gallic Acid Equivalents/g DM (mg GAE/g DM). Therefore, a particle size of 2–4.75 mm was selected for the following experiments.

Different solid-to-liquid ratios were studied: 1/20, 1/30, 1/40, 1/50, and 1/60. Decreasing the solid to liquid ratio from 1/20 to 1/50 g/mL increased the phenolic yield from 30 to 74 mg GAE/g DM. The same Total Phenolic Content (TPC) was observed for higher solid to liquid ratios. Therefore, 1/50 g/mL was adopted for the subsequent experiments.

### 2.2. Influence of Time, Temperature, and Ethanol Percentage on TPC Yield, and DPPH Inhibition Percentage

Optimization of polyphenols extraction was performed by the response surface methodology to select the optimal conditions for the highest extraction yield and antiradical activity for both WB and IR extraction techniques. A model was designed by setting the solid to liquid ratio to 1/50 (g/mL) and the particle size to 2–4.75 mm, varying time, temperature, and solvent mixture. TPC values (mg GAE/g DM) and DPPH inhibition percentages for WB and IR extracts are summarized in Table 1.

**Figure 1:**
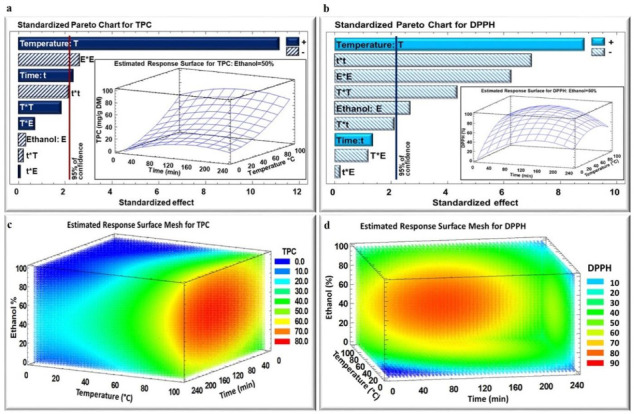
Standardized Pareto Chart for TPC (**a**) and DPPH (**b**) inhibition percentage for IR extraction, and estimated response surface Mesh for TPC (**c**) and DPPH (**d**) inhibition percentage. (+) indicates positive effect, (−) indicates negative effect.

**Figure 2:**
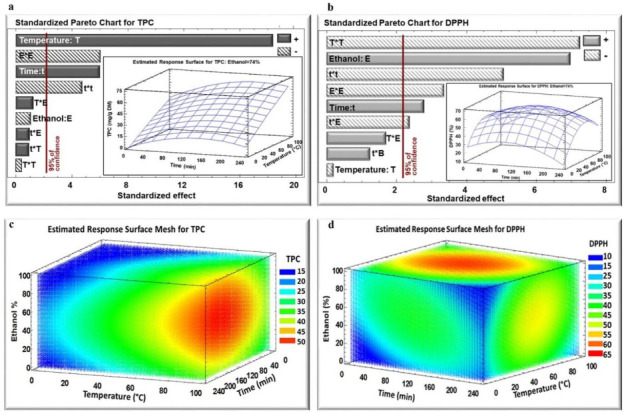
Standardized Pareto Chart for TPC (**a**) and DPPH (**b**) inhibition percentage for WB extraction, and estimated response surface Mesh for TPC (**c**) and DPPH (**d**) inhibition percentage. (+) indicates positive effect, (−) indicates negative effect.

The impact of the three studied parameters on the TPC and DPPH assay in dried *E. creticum* leaf extract was analyzed based on the Pareto chart (Figure 1 and Figure 2a,b) and estimated response surface Mesh (Figure 1 and Figure 2c,d) for both IR and WB extracts, respectively. Figure 1 and Figure 2a,b, show the Pareto Chart for the parameter’s extraction time, temperature, and ethanol percentage, including the estimated response surface. It was noticed that the temperature exhibited a significant positive linear effect on the polyphenol extraction yield from *E. creticum* leaves for both WB and IR extraction techniques, and a significant negative quadratic effect only in the case of WB for the DPPH inhibition percentage. In addition, extraction time had a positive linear effect on TPC for both WB and IR extraction techniques. On the other hand, the ethanol percentage revealed a negative quadratic effect on TPC and DPPH for both IR and WB extraction techniques.

Response values were given by statistical analysis to fit the second-degree regression model equation of the form shown in Table 2. The maximum TPC yield was obtained with an ethanol percentage of 50% and 75% for IR and WB, respectively.

### 2.3. Optimization of Extraction

Optimal extraction conditions were selected based on TPC and DPPH inhibition percentage of the obtained extracts. The IR technique was found to improve the extraction yield of polyphenols, by 1.7 times, compared to the conventional method (water bath extraction) from *E. creticum* leaves by increasing the TPC value from 45.5 mg GAE/g DM for WB extraction to 76 mg GAE/g DM for IR extraction (Table 3).

**Figure 3:**
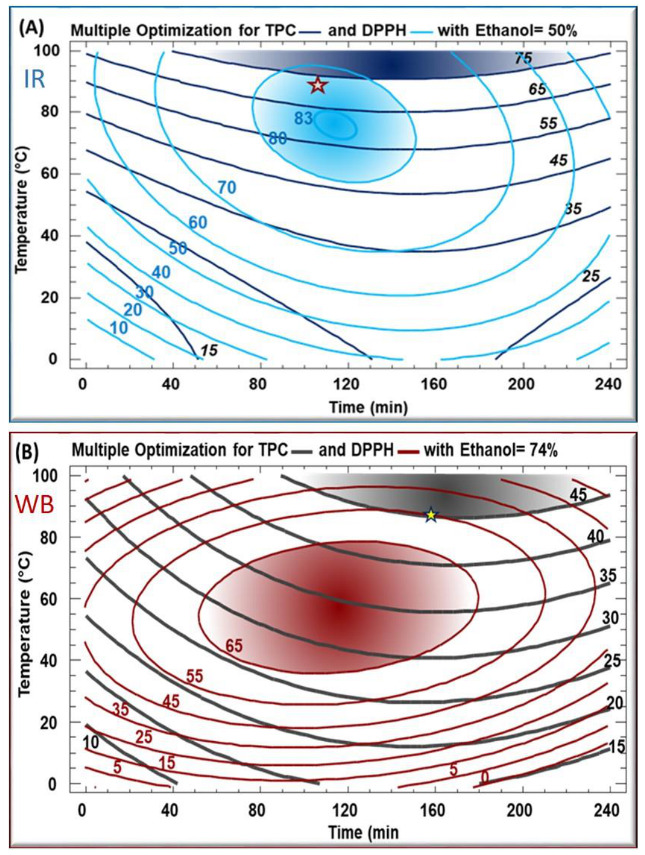
Contours plots generated from multiple response analysis for TPC and DPPH for *Eryngium creticum* IR (**A**) and WB (**B**) extracts. The star represents the multiple optimum conditions.

In order to maximize both the polyphenol quantity and their biological activity simultaneously, a multiple response optimization was applied in Figure 3, and the best experimental conditions were determined. Table 3 shows the optimal conditions for the recovery of TPC from both WB (167 min, 89 °C, 75% ethanol) and IR (130 min, 88 °C, 55% ethanol), as well as those for the obtainment of the highest antiradical activity. The model had a satisfactory level of adequacy with high R^2^, indicating a reasonable agreement of the corresponding model with the experimental results (Table 3).

Finally, the extraction using both methods was conducted under the predicted optimal conditions in order to verify the predictive capacity of the model. TPC and DPPH (Table 3) were tested and predicted values were confirmed by the experimental ones.

### 2.4. Identification of Polyphenols by UHPLC Analysis of the Optimal Conditions for IR and WB Extracts

The UHPLC analyses of both optimized *E. creticum* leaf extracts (IR and WB) were performed in order to assess the effect of the extraction technique on the phytochemical profile, and to correlate these results with the TPC values. The results showed that rutin and sinapic acid were the phenolic compounds detected in both extracts, with rutin being the most abundant identified compound. According to the results in Table 4, optimum conditions led to the highest values of rutin: 9.8 μg/mL and 6.4 μg/mL, for the IR and WB extracts, respectively. The IR extract had a threefold higher concentration of sinapic acid, compared to WB.

### 2.5. Antioxidant Activities of IR and WB Extracts Obtained under the Optimal Extraction Conditions

**Figure 4:**
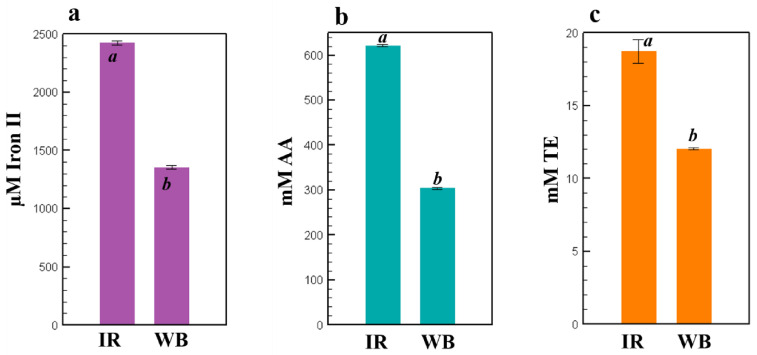
Antiradical and antioxidant capacities of *Eryngium creticum* leaf extracts were assessed by FRAP (**a**), ABTS (**b**), and CUPRAC (**c**). Letters a and b on the bars indicate significant statistical difference.

FRAP, ABTS, and CUPRAC assays were used to assess the antioxidant capacities of the optimized *E. creticum* leaf IR and WB extracts (Figure 4). The IR extract gave the highest antioxidant activity of 2424 ± 16.4 µM Iron II, 621.52 ± 2.13 mM AA, and 18.7 ± 0.81 mM TE compared to WB extracts of 1355 ± 16.4 µM Iron II, 303.76 ± 2.15 mM AA, and 12.0 ± 0.07 mM TE for FRAP, ABTS, and CUPRAC, respectively.

### 2.6. Antibacterial Activities of the Optimal Conditions for IR and WB Extracts

*E. creticum* leaf extracts showed an antibacterial activity against Gram-positive and Gram-negative tested bacterial strains. MIC and MBC values are summarized in Table 5.

*E. creticum* leaf extracts exhibited the highest antibacterial activity against the *S. epidermidis* strain with an MIC value of 50 mg/mL and 75 mg/mL for WB and IR extracts, respectively. Moreover, an MBC value of 75 mg/mL and 100 mg/mL for WB and IR extracts was shown, respectively. Nonetheless, the IR extract showed the highest antibacterial activity against *E. coli* (Gram-negative) with an MIC of 75 mg/mL, lower than the WB extract with an MIC value of 100 mg/mL. Furthermore, both *E. creticum* leaf extracts exhibited the same antibacterial activity against *P. aeruginosa* and *S. aureus* with an MIC value of 100 mg/mL.

### 2.7. Antibiofilm Prevention Activity of IR and WB Extracts Obtained at Optimal Extraction Condition

**Figure 5:**
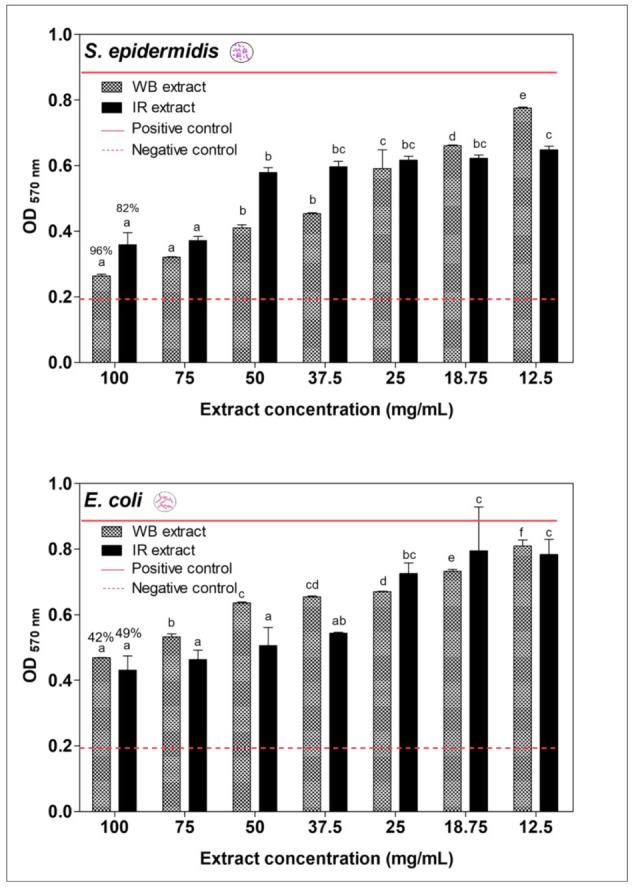
Preventing antibiofilm activity of different concentrations of *Eryngium creticum* leaf extracts on *Staphylococcus epidermidis* and *Escherichia coli* strains. Red continuous line corresponds to OD 0.9, red dashed line corresponds to OD 0.2. Different letters (a, b, c, d, e, and f) on the bars indicate significant statistical difference (*p* < 0.05).

Figure 5 shows the antibiofilm preventing the activity of *E. creticum* leaf extracts on *S. epidermidis* and *E. coli* bacterial strains using two extraction techniques (IR and WB), by representing the optical density of crystal violet (O.D _570 nm_) as a function of different extract concentrations (mg/mL).

To our knowledge, no studies have been reported about the antibiofilm prevention activity of *E. creticum* extracts. Results demonstrated that both extracts of *E. creticum* exhibited an antibiofilm activity against *S. epidermidis* (Gram-positive) and *E. coli* (Gram-negative) tested strains, with a strong antibiofilm activity against *S. epidermidis*.

The maximal biofilm prevention capacity against *S. epidermidis* was 96% and 82% for *E. creticum* for the WB and IR extracts, respectively, at a concentration of 100 mg/mL. Moreover, the WB extract exhibited a higher antibiofilm activity against *S. epidermidis* with a prevention capacity of 96% compared to the capacity of 42% against *E. coli*. However, the IR extract showed a higher antibiofilm activity against *S. epidermidis* with a prevention capacity of 82% compared to the capacity against *E. coli* of 49%.

## 3. Discussion

The reduction in particle size from a whole leaf to 2–4.75 mm increased the polyphenols content by 1.7 times in the extract. Particle size reduction is known to increase the extraction yield of phenolic compounds, since the augmentation of the contact surface enhances the accessible area for mass transfer between plant and solvent [13]. Concerning the determination of the solid-to-liquid ratio, the phenolic yield is increased while decreasing the solid-to-liquid ratio from 1/20 to 1/50 g/mL, and remained constant at a higher solid-to-liquid ratio than 1/50. Increasing the volume leads to higher diffusivity of target molecules into the extracting solvent [15]. For that reason, this solid-to-liquid ratio was adopted for the subsequent experiments.

After selecting the most suitable particle size (2–4.75 mm) and solid-to-liquid ratio (1/50 g/mL), the optimization process of polyphenols recovery was performed by the response surface methodology. It is noticeable that temperature and time had positive linear effects on TPC for both WB and IR since their increase led to the maximum TPC yield. Many studies have confirmed that increasing the temperature improves the extraction yield by improving the mass transfer through the enhancement of both solubility of solute and diffusion coefficients [15,16,17]. On the other hand, high temperatures are likely to break the cell walls releasing polyphenols. However, the negative quadratic effect of time on TPC is explained by the fact that with high extraction temperatures coupled with long periods of time, some phenolic compounds may be lost or degraded [8].

Temperature and time also had negative quadratic effects on the antiradical activity of both IR and WB extracts. In spite of the positive linear effect of higher extraction temperature on TPC yield, the heating process negatively affects the quality of the phenolic compounds subjecting them to degradation and/or oxidation [17,18,19].

The ethanol percentage had negative linear and quadratic effects on TPC yield and DPPH for IR. The increase in ethanol percentage affected negatively the IR efficiency, which is probably related to the high diversity of phenolic compounds, and their different solubility in water or ethanol [13,14]. This might be also due to the adequation between the absorption characteristics of water molecules with the used IR wavelengths. This observation suggests that the majority of the phenolic compounds were extracted using an intermediate solvent polarity varying between that of pure water and pure ethanol. Treatment by infrared irradiations showed a great impact on the improvement in TPC yield. The IR technique induced higher cellular and structural modifications leading to higher polyphenols release that may be due to the molecular rocking and twisting caused by the infrared irradiations [20]. The basic features of infrared radiation are the high capacity of heat transfer, and heat penetration directly into the product [21].

For WB extraction, despite the non-significant linear negative effect of ethanol percentage on TPC, there was a significant negative quadratic effect on TPC yield. Previous studies have proven the influence of an ethanol–water mixture on the phenolic compounds’ extraction compared to water [22,23].

The maximum TPC yield was obtained with an ethanol percentage of 50% and 75% for IR and WB, respectively (Table 2; Figure 3), which explains that the emitted IR wavelength is related to the solvent polarity [21]. Moreover, the reduction in organic solvent usage with IR limits environmental pollution, which is considered one of the most important concerns of green extraction.

Phenolic compounds were identified and quantified in both *E. creticum* leaf extracts by UHPLC analysis. The diversity and selectivity of extracted compounds could be related to the molecule’s structure as well as the extraction conditions of each method [24].

Many studies have reported on the presence of different phenolic compounds in *Eryngium* extracts [25,26,27,28]. Our results are in agreement with the literature, which reported that among the phenolic compounds, rutin and sinapic acid were determined in *E. planum*, *E. campestre,* and *E. maritimum* extracts [25,26]. Likewise, previous studies confirmed that rutin is the major phenolic compound with a concentration of 290.5 μg/mL in *E. planum* [25] compared to 9.8 μg/mL and 6.4 μg/mL for *E. creticum* IR and WB extracts, respectively.

The phenolic compounds exhibited a wide range of biological activities including antioxidant, anti-inflammatory, antibacterial, antiviral, and therapeutic, leading to pharmaceutical, cosmetic, and food industry applications [29]. Furthermore, the usage of these bioactive molecules in various industrial or pharmaceutical applications in food supplements is very important. Therefore, the presence of these valuable phenolic compounds in *Eryngium* extracts might be responsible for many health benefits, and could explain the biological activity of *Eryngium* leaf extracts. In the literature, rutin is reported as an antimicrobial, antifungal, anti-inflammatory, and anticancer agent, as well as a potent antioxidant compound [30]. Moreover, sinapic acid is a phytochemical found in various plants recognized for their antibacterial and antioxidant capacities [31,32]. Several studies conducted on various Gram-positive and Gram-negative bacterial strains indicated the significant antibacterial potential of sinapic acid [32].

Concerning the antiradical and antioxidant capacities, *E. creticum* leaf IR extract gave the highest activity compared to the WB extract. Therefore, IR treatment proved to intensify TPC extraction yield from *E. creticum* leaves while preserving the antioxidant-antiradical capacities. A previous study has reported the antioxidant capacity of ethanolic *E. creticum* extract obtained by maceration for 48 h, with a 15% inhibition of DPPH [33]. This inhibition percentage is lower than the 63% and 83% obtained for the WB and IR *E. creticum* leaf extracts in this study, respectively.

Our results showed that *E. creticum* leaf WB extract exhibited the highest antibacterial activity against *S. epidermidis* with an MIC value of 50 mg/mL; while *E. creticum* leaf IR extract showed the highest antibacterial activity against *E. coli* with an MIC value of 75 mg/mL; this may be explained by the selectivity of the extraction method on the extracted bioactive compounds. A previous study has reported the antibacterial activity of aqueous and ethanolic extracts of *E. creticum* leaves, extracted by maceration for 8 h at 37 °C, against Gram-positive and Gram-negative bacteria, with an MIC of 354 mg/mL and 294 mg/mL for aqueous and ethanolic extracts, respectively, against *S. aureus*; however, *E. coli* was highly resistant and not inhibited even at highest tested concentration [34]. This demonstrates the efficiency of the IR technique by lowering the minimal inhibition concentration to 100 mg/mL against the *S. aureus* strain. Meot-Duros et al. have reported on the antibacterial activity of *E. maritimum* leaf extract against *S. aureus*, *P. aeruginosa*, *E. coli,* and other bacterial strains, with the highest sensitivity being of *P. aeruginosa* [35].

## 4. Materials and Methods

### 4.1. Plant Material

*Eryngium creticum* was collected from the South Lebanon region at about 400 m of altitude. It was identified by referring to the well-known guide of Lebanon’s flora [36]. Before the extraction process, the plant’s materials were cleaned and dried in an airflow oven (UFE 700, Memmert GmbH, Schwabach, Germany) at 35 °C for 48 h. Dried leaves were ground using a grinder with a blade (IKA knife mill M20) and sieved to a particle size of 2–4.75 mm, and stored in the dark at room temperature for further use.

### 4.2. Chemicals, Media, and Bacterial Strains

Folin–Ciocalteu reagent, sodium carbonate, gallic acid (3,4,5-trihydroxybenzoic acid), DPPH (2,2-diphenyl-picrylhydrazyl), Trolox (6-hydroxy-2,5,7,8-tetramethylchromane-2-carboxylic acid), as well as all HPLC standards and solvents were purchased from Sigma-Aldrich (Steinheim, Germany).

Tryptic Soy Broth (TSB), Luria-Bertani broth (LB), Brain Heart Agar (BHA), and Mueller–Hinton Broth (MHB) were purchased from HIMEDIA (Mumbai, India). All media were prepared in accordance with the manufacturer’s instructions.

Two Gram-positive strains and two Gram-negative strains (Table 6) were used to determine the antibacterial activity of *E. creticum* extracts. All strains were stored at −80 °C in glycerol before use.

### 4.3. Dry Matter Content

Dry matter (DM) content of *E. creticum* was determined by drying the leaves in a ventilated oven at 105 °C for 24 h, and the DM content was calculated and expressed in percent (% *w*/*w*). The DM is 88 ± 0.006% *w*/*w*.

### 4.4. Extraction Procedures

A preliminary study was conducted using different particle sizes (whole leaf, 6.5–4.75 mm, 2–4.75 mm) and different solid-to-liquid ratios (1/20 to 1/60 g/mL). After defining the particle size and the solid-to-liquid ratio, the optimization of the extraction process was performed using RSM. Two grams of dried *E. creticum* leaves were added to 100 mL of solvent (solid to liquid ratio of 1/50) with various percentages of ethanol–water mixture *v*/*v* (from 16% to 84%). The extracts were filtered, and the filtrates were condensed by evaporation using a rotary evaporator (Heidolph, Germany). Subsequently, the filtrates were frozen at −80 °C prior to lyophilization (Freezer dryer, Alpha 1-4 LD plus, Germany).

#### 4.4.1. Water Bath Extraction (WB)

**Figure 6:**
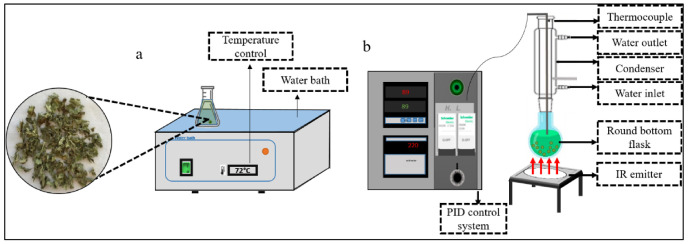
Experimental setup of water bath (**a**) and Infrared (**b**) equipment.

The Water Bath extraction (WB) was carried out using a water bath (Clifton, UK) as illustrated in Figure 6a. Dried *E. creticum* leaves (size of 2–4.75 mm) were placed in an Erlenmeyer with the solvent mixture at the appropriate temperature for a given extent of time with stirring.

#### 4.4.2. Infrared-Assisted Extraction (IR)

The IR extraction was conducted using the apparatus illustrated in Figure 6b. The extraction prototype is based on a ceramic infrared emitter (Rotfil, Pianezza, Italy) for the heating process, linked to a proportional–integral–derivative (PID) control for temperature adjustment. Dried *E. creticum* leaves (size of 2–4.75 mm) were placed in a round bottom flask with the solvent mixture, connected to a condenser, at the appropriate temperature for a given time. The round bottom flask was maintained at a 1 cm distance from the IR emitter.

### 4.5. Experimental Design

The quality and quantity of the phenolic compounds are affected by several parameters. The optimization of the extraction process was performed using RSM, taking into consideration “t” as the extraction time, “T” as the extraction temperature, and “E” as the solvent mixture, to evaluate the effect of each parameter and the interaction between parameters. A rotatable central composite design (2^3^ + star), with 22 runs including 8 repetitions at the central points, was established to assess the main effect of these 3 factors on TPC and DPPH inhibition percentage as response parameters. The extraction time varied between 70 and 170 min, the temperature varied between 35 and 75 °C, and the ethanol percentage varied between 30% and 70% *v*/*v*. The used higher and lower bonds were considered as −1 and +1 levels, respectively. The same design was used both for WB and IR extraction techniques.

Considering three parameters and one response, experimental data were fitted to obtain a second-degree regression equation of the form:

Y = α_0_ + α_1_ t + α_2_ T + α_3_ E + α_4_ t^2^ + α_5_ T^2^ + α_6_ E^2^+ α_7_ t T + α_8_ t E + α_9_ T E

where “Y” is the predicted response parameter; α_0_ is the mean value of responses at the central point of the experiment; α_1_, α_2_, and α_3_ are the linear coefficients; α_4_, α_5_, and α_6_ are the quadratic coefficients; α_7_, α_8_, and α_9_ are the interaction coefficients. Experimental design and statistical analysis of the results were performed using STATGRAPHICS Centurion XVII-X64.

### 4.6. Determination of TPC

The TPC was determined according to the Folin–Ciocalteu method previously described by Slinkard and Singleton [37]. A volume of 200 μL of the *E. creticum* extract was mixed with 1000 μL of Folin–Ciocalteu reagent (diluted 1/10 *v*/*v*) and 800 μL of Na_2_CO_3_ 7.5% (*w/v*). The blend was incubated for 10 min at 60 °C and 10 min at 4 °C. The absorbance was measured at 750 nm using a UV–Vis spectrophotometer (GENESYS 10 UV, Thermo Electron Corporation, Waltham, MA, USA). Gallic acid was used as standard for the calibration curve determination. The TPC was expressed as mg of gallic acid equivalents per gram of dry matter (mg GAE/g DM).

### 4.7. Ultra–High–Performance Liquid Chromatography (UHPLC)

Phenolic compounds in the *E. creticum* extracts were separated using a Thermo Vanquish UHPLC system (Thermo Scientific, Waltham, MA, USA) equipped with a pump, degasser, autosampler, and a photodiode array detector (PDA). Samples (5 μL) were injected onto an Acquity BEH C18 column (150 × 2.1 mm, particle size 1.7 μm) (Waters, Milford, MA, USA). The flow rate used was 0.350 mL/min at a column temperature of 25 °C. The eluents used were formic acid (0.1% *v*/*v*) in water (A) and acetonitrile (B). The following gradient was used: 0–2 min at 4% B (isocratic), 2–30 min from 4 to 60% B (linear gradient), 30–30.1 min 60 to 100% B (linear gradient), 30.1–35 min at 100% B (isocratic), 35–35.1 min from 100 to 4% B (linear gradient), and 35.1–40 min at 4% B (isocratic). The PDA detector was set to record wavelengths between 200 and 680 nm. Identification of the compounds was performed by comparison of retention time and spectra with representative standards. Quantification of phenolic compounds was achieved by construction of calibration curves of rutin, caffeic acid, sinapic acid, vanillic acid, ferulic acid, and coumaric acid.

### 4.8. Antiradical Activity

Antiradical activity was measured by the capacity of the phenolic compounds in the samples to reduce the free radical, DPPH (2,2-diphenyl-picrylhydrazyl) [38]. Briefly, 1.45 mL of DPPH (0.06 mM) radical was added to 50 μL of *E. creticum* extracts or Trolox (as positive control). The absorbance at 515 nm was measured after 30 min of incubation in the dark at room temperature using pure methanol as a blank. The inhibition percentage of the DPPH free radical is calculated as follows:

$$Inhibition\;percentage\;=\frac{Abs\;of\;negative\;control-Abs\;of\;sample}{Abs\;of\;negative\;control}\;\times 100$$

Antiradical activity was expressed as μg of Trolox equivalent per milliliter (μg TE/mL).

### 4.9. Antioxidant Activity

#### 4.9.1. Ferric Reducing Antioxidant Power Assay (FRAP)

This assay was completed using the FRAP antioxidant capacity kit (Bioquochem, Asturias, Spain), which measures the antioxidant activity of compounds that are able to reduce the ferric complex at an acidic pH, in the presence of a suitable antioxidant solution. Briefly, 10 μL of the extracts, or standard, was added to 220 μL of ready-to-use FRAP working solution. The absorbance was measured using a plate reader at 593 nm after 4 min mixing under continuous stirring. The antioxidant activity was expressed as μM of Iron (II) equivalent (Iron (II) μM).

#### 4.9.2. 2,2′-Azinobis (3-Ethylbenzothiazoline-6-Sulphonic Acid) Radical Scavenging Assay (ABTS)

The ABTS assay was conducted using the ABTS Assay Kit (Bioquochem, Asturias, Spain). An amount of 200 μL of previously prepared ABTS solution was mixed with 5 μL of the diluted extracts or standard (ascorbic acid). The absorbance was monitored spectrophotometrically using a plate reader at 734 nm after 5 min under continuous stirring. The ABTS scavenging capacity was expressed in mM of ascorbic acid equivalent (AA mM).

#### 4.9.3. Cupric Ion Reducing Antioxidant Capacity Assay (CUPRAC)

CUPRAC assay kit (Bioquochem, Asturias, Spain) was also applied to measure the antioxidant activity of the extract. CUPRAC enables measurements of the total antioxidant capacity by the oxidation of the copper (II)-neocuproine (2,9-dimethyl-1,10-phenanthroline). An amount of 40 μL of the diluted extracts, or standard (Trolox), was added to 200 μL of previously prepared working solution. The mixture was incubated at room temperature for 30 min, and then the absorbance was measured at 450 nm using a plate reader. Results were expressed as mM Trolox Equivalent (TE mM).

### 4.10. Minimal Inhibitory Concentration (MIC) and Minimal Bactericidal Concentration (MBC) Assays

MICs and MBCs were determined using the microdilution method as recommended by the Clinical and Laboratory Standards Institute (CLSI) adopted in our RLM [39,40]. A total of 100 µL of each extract was used to perform serial twofold dilutions in MHB using a 96-well cell culture plate. Then, a diluted bacterial suspension (volume of 5 μL) was added to each well to give a final concentration of 5 × 10^5^ CFU/mL (according to CLSI recommendation). Wells without any plant extract but with bacterial inoculum were used as a positive control and those without bacterial inoculum as negative control. The plates were incubated at 37 °C for 24 h. The MIC of each extract was determined by observing the lowest concentration of extract where no visible growth was observed compared to the negative and positive controls. Afterward, the contents of all wells with no visible growth were plated on BHA in order to determine the MBC, which is the lowest concentration of each extract killing all the bacterial population by >99.9% (<5 CFU/plate).

### 4.11. Biofilm Prevention Assay

A 96-well polystyrene tissue culture-treated microtiter plate was used to assess the biofilm prevention activity of *E. creticum* extracts. A total of 100 µL of TSB medium supplemented with 0.25% (*w*/*v*) glucose and 100 µL of extracts were added to the first well of microplates and serial twofold dilution was performed. A diluted bacterial suspension (5 µL) was added as inoculum to each well to give a final concentration of 5 × 10^5^ CFU/mL. Wells without any plant extract were used as a positive control for biofilm formation and wells without bacterial inoculum were used as a negative control. The microplate was incubated at 37 °C for 24 h. Hereafter, the well content was removed, and the biofilm formed in the wells was fixed by heating for 1 h at 80 °C. Then, 100 µL of crystal violet (0.1%) was added to each well for staining and subsequently washed after 5 min. Finally, 100 µL of distilled water was added and the optical density (OD) was measured at 570 nm using a microplate reader (Tristar2 S LB 942, Berthold, Germany) [41,42]. Minimal biofilm prevention concentration (MBPC) was defined as being the lowest concentration exhibiting the highest significant biofilm formation prevention.

### 4.12. Statistical Analysis

All experiments and measurements were performed in triplicate (n = 3) and the results were expressed as mean value ± standard deviation. The data between the control and experimental groups were analyzed by *p* values; *p*-values < 0.05 were considered significant with a more than 95% confidence rate. Results were analyzed by variance analyses (ANOVA) and the statistical analyses were carried out using STATGRAPHICS^®^ Centurion XVII-X64 software for extraction optimization process and GraphPad Prism^®^ Software (Version 6.05; GraphPad Software, Inc., San Diego, CA, USA) for prevention of antibiofilm activity.

## 5. Conclusions

This study demonstrated the efficiency of infrared irradiations in intensifying the extraction of phenolic compounds from dried *E. creticum* leaves. Compared to water bath extraction, IR-assisted extraction improved the polyphenols recovery by 1.7 times, while shortening the extraction time and decreasing ethanol consumption by 1.5 times. This improvement was also confirmed by UHPLC results, in which the same phenolic compounds in the WB extract were detected at higher concentrations after IR extraction. Both extracts of *E. creticum* leaves exhibited a strong antibiofilm activity against *S. epidermidis*. *Ired-Irrad^®^* is a new extraction technique that can enhance polyphenol extraction with less extraction time and solvent consumption, while preserving their quality as compared to those obtained by a conventional technique.

## 6. Patent

Rajha, H.N.; Debs, E.; Maroun, R.G.; Louka, N. Système d’extraction, de séparation ou de prétraitement assisté par rayonnement infrarouge. Adéquation entre les caractéristiques du rayonnement et celles de la matière traitée. Lebanese Patent 2017/11-11296L, granted on 29 November 2017.

## Figures and Tables

**Table 1:** Central composite arrangement for independent variables and their responses for TPC (mg GAE/g DM) and DPPH inhibition percentage.

Run	Variable			Responses			
	Time (min)	Temperature (°C)	Ethanol Percentage (%)	IR		WB	
				TPC (mg GAE/g DM)	DPPH Inhibition Percentage	TPC (mg GAE/g DM)	DPPH Inhibition Percentage
1	70	35	30	23.39	45.15	17.46	25.45
2	170	35	30	26.08	53.10	19.42	38.93
3	70	75	30	44.59	81.80	35.56	7.42
4	170	75	30	47.73	77.71	40.87	28.65
5	70	35	70	18.13	33.53	12.55	61.53
6	170	35	70	23.04	44.70	17.99	41.21
7	70	75	70	45.23	65.50	35.02	56.51
8	170	75	70	47.14	55.04	43.14	56.96
9	36	55	50	30.69	41.41	20.19	11.98
10	204	55	50	44.93	55.29	38.29	53.05
11	120	21	50	26.28	34.95	15.68	16.21
12	120	88	50	73.53	83.20	57.20	23.17
13	120	55	16	35.41	50.33	28.36	14.52
14	120	55	83	37.41	52.26	25.76	70.43
15	120	55	50	39.42	80.81	36.42	66.71
16	120	55	50	39.17	80.84	35.97	69.63
17	120	55	50	40.93	80.46	35.51	67.09
18	120	55	50	40.58	80.36	36.40	67.81
19	120	55	50	39.42	80.94	34.68	68.15
20	120	55	50	40.48	79.19	35.27	68.72
21	120	55	50	40.82	78.42	34.23	69.63
22	120	55	50	40.73	79.71	34.68	67.33

**Table 2:** Second-order regression equations for IR and WB extraction.

Extraction Technique	Equations
**IR**	**TPC** = − 10.21 + 0.29 t + 0.0015 T + 0.52 E − 0.0009 t^2^ − 0.0003 t T + 0.0001 t E + 0.005 T^2^ + 0.003 T E − 0.007 E^2^ **DPPH** = − 159.174 + 1.25 t + 3.19 T + 2.41 E − 0.004 t^2^ − 0.004 t T − 0.0004 t E − 0.015 T^2^ − 0.0056 T E − 0.02 E^2^
**WB**	**TPC** = − 26.83 + 0.26 t + 0.42 T + 0.62 E − 0.00111123 t^2^ + 0.0007 t T + 0.0008 t E − 0.0006 T^2^ + 0.002 T E − 0.009 E^2^ **DPPH** = − 184.92 + 1.27 t + 3.06 T + 2.65 E− 0.004 t^2^ + 0.0035 t T− 0.007 t E − 0.03 T^2^ + 0.012 T E − 0.017 E^2^

**Table 3:** Optimum extraction conditions for WB and IR techniques.

Parameters	Optimum Conditions			
	WB		IR	
	TPC	DPPH	TPC	DPPH
Time (min)	167	116	130	113
Temperature (°C)	89	58	88	79
Ethanol (%)	75	74	55	42
TPC optimal value (mg GAE/g DM)	56.6		77	
DPPH optimal value (%)		76		88.8
R-squared	97.3	92.65	92.4	94.17
**Parameters**	**Multiple Optimization**			
	**WB**		**IR**	
Time (min)	162		109	
Temperature (°C)	91		89	
Ethanol (%)	75		50	
TPC value predicted (mg GAE/g DM)	45.5		76	
TPC value observed (mg GAE/g DM)	44.60 ± 1.6		75.83 ± 1.3	
DPPH inhibition percentage predicted (%)	66.2		83.7	
DPPH inhibition percentage observed (%)	63.10 ± 1.3		82.80 ± 0.5	

**Table 4:** Phenolic compounds concentrations (μg/mL) in IR and WB *Eryngium creticum* leaf extracts.

Extraction Method	Concentration (μg/mL)	
	Rutin	Sinapic Acid
**IR**	9.8	4
**WB**	6.4	1.3

**Table 5:** MIC and MBC values of *Eryngium creticum* leaf extracts against *Escherichia coli*, *Pseudomonas aeruginosa*, *Staphylococcus epidermidis*, and *Staphylococcus aureus*.

Plant extract	MIC (mg/mL)				MBC (mg/mL)			
	*E. coli*	*P. aeruginosa*	*S. epidermidis*	*S. aureus*	*E. coli*	*P. aeruginosa*	*S. epidermidis*	*S. aureus*
IR	75	100	75	100	100	>100	100	>100
WB	100	100	50	100	100	100	75	100

(>) indicates that a higher concentration of extract might be needed to achieve the antibacterial effect.

**Table 6:** Bacterial strains used in this study.

	Bacterial Strains	Reference
**Gram-positive**	*Staphylococcus aureus*	ATCC 49619
	*Staphylococcus epidermidis* RP62A	ATCC 35984
**Gram-negative**	*Escherichia coli*	ATCC 35218
	*Pseudomonas aeruginosa*	ATCC 27853

## Data Availability

Not applicable.

## References

[1] Küpeli E., Kartal M., Aslan S., Yesilada E. (2006). Comparative Evaluation of the Anti-Inflammatory and Antinociceptive Activity of Turkish Eryngium Species. J. Ethnopharmacol..

[2] Kikowskaa M., Dworacka M., Kędziora I., Thiem B. (2016). Eryngium Creticum – Ethnopharmacology, Phytochemistry and Pharmacological Activity. A Review. Braz. J. Pharmacogn..

[3] Kartal M., Mitaine-Offer A.-C., Abu-Asaker M., Miyamoto T., Calis I., Wagner H., Lacaille-Dubois M.-A. (2005). Two New Triterpene Saponins from Eryngium Campestre. Chem. Pharm. Bull..

[4] Wang P., Su Z., Yuan W., Deng G., Li S. (2012). Phytochemical Constituents and Pharmacological Activities of Eryngium L. (Apiaceae). Pharm. Crop..

[5] Erdelmeier C.A.J., Sticher O. (1985). Coumarin Derivatives from Eryngium Campestre. Planta Med..

[6] Cory H., Passarelli S., Szeto J., Tamez M., Mattei J. (2018). The Role of Polyphenols in Human Health and Food Systems: A Mini-Review. Front. Nutr..

[7] Aires A., Soto-Hernandez M., Palma-Tenango M., Garcia-Mateos M.d.R. (2017). Phenolics in Foods: Extraction, Analysis and Measurements. Henolic Compounds—Natural Sources, Importance and Applications.

[8] Azmir J., Zaidul I.S.M., Rahman M.M., Sharif K.M., Mohamed A., Sahena F., Jahurul M.H.A., Ghafoor K., Norulaini N.A.N., Omar A.K.M. (2013). Techniques for Extraction of Bioactive Compounds from Plant Materials: A Review. J. Food Eng..

[9] Khoddami A., Wilkes M.A., Roberts T.H. (2013). Techniques for Analysis of Plant Phenolic Compounds. Molecules.

[10] Rajha H.N., Debs E., Maroun R.G., Louka N. (2017). Système d’extraction, de Séparation Ou de Prétraitement Assisté Par Rayonnement Infrarouge. Adéquation Entre Les Caractéristiques Du Rayonnement et Celles de La Matière Traitée. Lebanese Patent.

[11] Abi-Khattar A.-M., Rajha H.N., Abdel-Massih R.M., Maroun R.G., Louka N., Debs E. (2019). Intensification of Polyphenol Extraction from Olive Leaves Using Ired-Irrad^®^, an Environmentally-Friendly Innovative Technology. Antioxidants.

[12] Escobedo R., Miranda R., Martínez J. (2016). Infrared Irradiation: Toward Green Chemistry: A Review. Int. J. Mol. Sci..

[13] Rajha H.N., Mhanna T., El Kantar S., El Khoury A., Louka N., Maroun R.G. (2019). Innovative Process of Polyphenol Recovery from Pomegranate Peels by Combining Green Deep Eutectic Solvents and a New Infrared Technology. LWT Food Sci. Technol..

[14] El Kantar S., Rajha H.N., Maroun R.G., Louka N. (2020). Intensification of Polyphenols Extraction from Orange Peels Using Infrared as a Novel and Energy Saving Pretreatment. Food Eng. Mater. Sci. Nanotechnol. Sci. Nanotechnol..

[15] Pinelo M., Rubilar M., Jerez M., Sineiro J., Núñez M.J. (2005). Effect of Solvent, Temperature, and Solvent-to-Solid Ratio on the Total Phenolic Content and Antiradical Activity of Extracts from Different Components of Grape Pomace. J. Agric. Food Chem..

[16] Spigno G., Tramelli L., Faveri D.M. (2007). De Effects of Extraction Time, Temperature and Solvent on Concentration and Antioxidant Activity of Grape Marc Phenolics. J. Food Eng..

[17] Rajha H.N., El Darra N., Vorobiev E., Louka N., Maroun R.G. (2013). An Environment Friendly, Low-Cost Extraction Process of Phenolic Compounds from Grape Byproducts. Optimization by Multi-Response Surface Methodology. Food Nutr. Sci..

[18] Cacace J.E., Mazza G. (2003). Mass Transfer Process during Extraction of Phenolic Compounds from Milled Berries. J. Food Eng..

[19] Rajha H.N., El Darra N., Hobaika Z., Boussetta N., Vorobiev E., Maroun R.G., Louka N. (2014). Extraction of Total Phenolic Compounds, Flavonoids, Anthocyanins and Tannins from Grape Byproducts by Response Surface Methodology. Influence of Solid-Liquid Ratio, Particle Size, Time, Temperature and Solvent Mixtures on the Optimization Process. Food Nutr. Sci..

[20] Chen Y., Duan G., Xie M., Chen B., Li Y. (2010). Infrared-Assisted Extraction Coupled with High-Performance Liquid Chromatography for Simultaneous Determination of Eight Active Compounds in Radix Salviae Miltiorrhizae. J. Sep. Sci..

[21] Richardson P. (2001). Thermal Technologies in Food Processing.

[22] Baldosano H., Castillo M.G., Elloran C., Bacani F.T. (2015). Effect of Particle Size, Solvent and Extraction Time on Tannin Extract from Spondias Purpurea Bark Through Soxhlet Extraction. Proc. DLSU Res. Congr..

[23] Dent M., Uzelac V., Penić M., Brnčić M., Bosiljkov T., Levaj B. (2012). The Effect of Extraction Solvents, Temperature and Time on the Composition and Mass Fraction of Polyphenols in Dalmatian Wild Sage (*Salvia officinalis* L.) Extracts. Food Technol. Biotechnol..

[24] Cai Y., Yu Y., Duan G., Li Y. (2011). Study on Infrared-Assisted Extraction Coupled with High Performance Liquid Chromatography (HPLC) for Determination of Catechin, Epicatechin, and Procyanidin B2 in Grape Seeds. Food Chem..

[25] Paun G., Neagu E., Moroeanu V., Albu C., Savin S., Lucian Radu G. (2019). Chemical and Bioactivity Evaluation of Eryngium Planum and Cnicus Benedictus Polyphenolic-Rich Extracts. Biomed Res. Int..

[26] Conea S., Vlase L., Chirila I. (2016). Comparative Study on the Polyphenols and Pectin of Three Eryngium Species and Their Antimicrobial Activity. Cellul. Chem. Technol..

[27] Kartal M., Mitaine-Offer A.-C., Abu-Asaker M., Miyamoto T., Calis. I., Wagner H. (2005). Phytochemical Constituents and Pharmacological Activities of Eryngium L. (Apiaceae). Planta Med..

[28] Elsbaey M., Ibrahim M.A.A., Shawky A.M., Miyamoto T. (2022). Eryngium Creticum L.: Chemical Characterization, SARS-CoV-2 Inhibitory Activity, and In Silico Study. ACS Omega.

[29] Maroun R.G., Rajha H.N., El Darra N., El Kantar S., Chacar S., Debs E., Vorobiev E., Louka N. (2018). Emerging Technologies for the Extraction of Polyphenols from Natural Sources.

[30] Gullón B., Lú-Chau T.A., Moreira M.T., Lema J.M., Eibes G. (2017). Rutin: A Review on Extraction, Identification and Purification Methods, Biological Activities and Approaches to Enhance Its Bioavailability. Trends Food Sci. Technol..

[31] Martinović N., Abramovi H. (2014). Sinapic Acid and Its Derivatives: Natural Sources and Bioactivity. Compr. Rev. Food Sci. Food Saf..

[32] Amarowicz R., Fornal J., Karamac M. (1992). Antioxidative and Bactericidal Properties of Phenolic Compounds in Rapeseeds. Eur. J. Lipid Sci. Technol..

[33] Damaj R., Sabbah A., Nasser G., Francis M.B., Hijazi A., Annan H., Al Rekaby A.A.A., Rammal H. (2016). Antioxidant Activity and Chemical Composition of the Ethanolic Extract from Leaves and Stems of the Lebanese *E. Cretcium*. J. Multidiscip. Eng. Sci. Technol..

[34] Makki R., Ze D., Rammal H., Sweidan A., Al Bazzal A., Chokr A. (2016). Antibacterial Activity of Two Lebanese Plants: Eryngium Creticum and Centranthus Longiflorus. Nanomedicine Nanotechnol..

[35] Meot-Duros L., Le Floch G., Magné C. (2008). Radical Scavenging, Antioxidant and Antimicrobial Activities of Halophytic Species. J. Ethnopharmacol..

[36] Tohmé G., Tohmé H. (2007). Illustrated Flora of Lebanon.

[37] Slinkard K., Singleton V. (1977). Total Phenol Analysis: Automation and Comparison with Manual Methods. Am. J. Enol. Vitic..

[38] Kallithraka S., Mohdaly A.A.A., Makris D.P., Kefalas P. (2005). Determination of Major Anthocyanin Pigments in Hellenic Native Grape Varieties (*Vitis vinifera* sp.): Association with Antiradical Activity. J. Food Compos. Anal..

[39] Clinical and Laboratory Standards Institute (2006). Clinical and Laboratory Standards Institute. Methods for Dilution Antimicrobial Susceptibility Tests for Bacteria That Grow Aerobically; Approved Standard.

[40] Kanaan H., El-Mestrah M., Sweidan A., As-Sadi F., Al Bazzal A., Chokr A. (2017). Screening for Antibacterial and Antibiofilm Activities in Astragalus Angulosus. J. Intercult. Ethnopharmacol..

[41] Christensen G.D., Simpson W.A., Younger J.J., Baddour L.M., Barrett F.F., Melton D.M., Beachey E.H. (1985). Adherence of Coagulase-Negative Staphylococci to Plastic Tissue Culture Plates: A Quantitative Model for the Adherence of Staphylococci to Medical Devices. J. Clin. Microbiol..

[42] Chokr A., Watier D., Eleaume H., Pangon B., Ghnassia J.-C., Dietrich M., Jabbouri S. (2006). Correlation between Biofilm Formation and Production of Polysaccharide Intercellular Adhesin in Clinical Isolates of Coagulase-Negative Staphylococci. Int. J. Med. Microbiol..

